# Low temperature enantiotropic nematic phases from V-shaped, shape-persistent molecules

**DOI:** 10.3762/bjoc.5.73

**Published:** 2009-12-04

**Authors:** Matthias Lehmann, Jens Seltmann

**Affiliations:** 1 Institute of Chemistry, Chemnitz University of Technology, Straße der Nationen 62, 09111 Chemnitz, Germany

**Keywords:** biaxial nematics, liquid crystals, phase engineering, thiadiazoles, V-shaped mesogens

## Abstract

A series of V-shaped, shape-persistent thiadiazole nematogens, based on an oligo(phenylene ethynylene) scaffold with ester groups connected via alkyloxy spacers, was efficiently prepared by a two-step procedure. Phase engineering results in an optimum of the mesophase range and low melting temperature when the nematogens are desymmetrised with a butoxy and a heptyloxy spacer. The mesophases are enantiotropic and over the whole temperature range nematic. For the optimised mesogen structure, optical investigations by conoscopy monitored a uniaxial nematic phase upon cooling from the isotropic phase to room temperature (Δ*T* = 150 °C). X-ray studies on magnetic-field-aligned samples of this mesogen family revealed a general pattern, indicating the alignment of two molecular axes along individual directors in the magnetic field. These observations may be rationalised with larger assemblies of V-shaped molecules isotropically distributed around the direction of the magnetic field.

## Introduction

**Figure 1:**
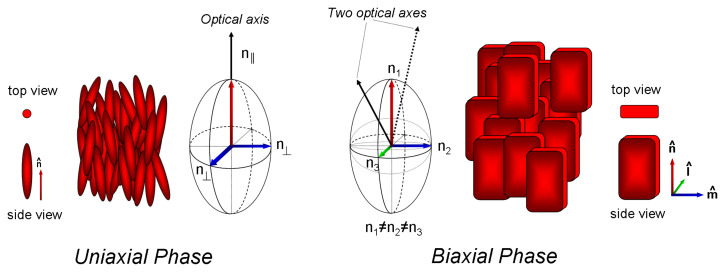
Uniaxial nematic (left) and biaxial nematic (right) phases and their corresponding indicatrices.

Most molecules forming nematic liquid crystals, the nematogens, are based on rod-shaped (calamitic), anisometric cores with peripheral flexible chains along the molecular long axis [1]. Nematic phases are the simplest liquid crystalline mesophases, in which phase anisotropy of crystals is combined with fluid properties of liquids. In the nematic phases of calamitic mesogens only the molecular long axes are oriented along a so called director [2] and the molecular centres of gravity are distributed like in a liquid. In models, the molecules are thought to turn rapidly about their long axis and therefore the shape of nematogens in theoretical studies has been simplified to a spherocylinder [3]. These phases are called uniaxial. Almost 40 years ago, M. J. Freiser predicted that real nematogens possess a non-cylindrical shape and thus should be able to form biaxial nematic phases at an appropriate low temperature [4]. What is a biaxial nematic phase and why is it so interesting? Uniaxial and biaxial phases can be best understood by the property from which this classification originates: the behaviour when light propagates in the material. The optical property of a uniaxial phase in a monodomain can be described by two refractive indices spanning a rotationally symmetric ellipsoid, also called indicatrix (Figure 1, left side). There is one particular direction, perpendicular to the circular cross section of this special ellipsoid, along which the propagating linear polarised light does not change its polarisation. This direction is called the optical axis. In a uniaxial phase there is only one optical axis. However, if the monodomains of materials have to be optically described with three different refractive indices, the indicatrix is an ellipsoid spanned by three different semi-principal axes, possessing two different circular cross sections and consequently two optical axes (Figure 1, right side).

In a biaxial nematic phase three molecular axes align along individual directors resulting in a material with three different refractive indices. However, at the same time this material has a liquid-like distribution of the molecular centres of gravity. In spite of the high molecular mobility the high order should be maintained. These two parameters have to be balanced extremely precise in order to obtain a thermotropic biaxial nematic with molecules of low molar mass [5].

**Figure 2:**
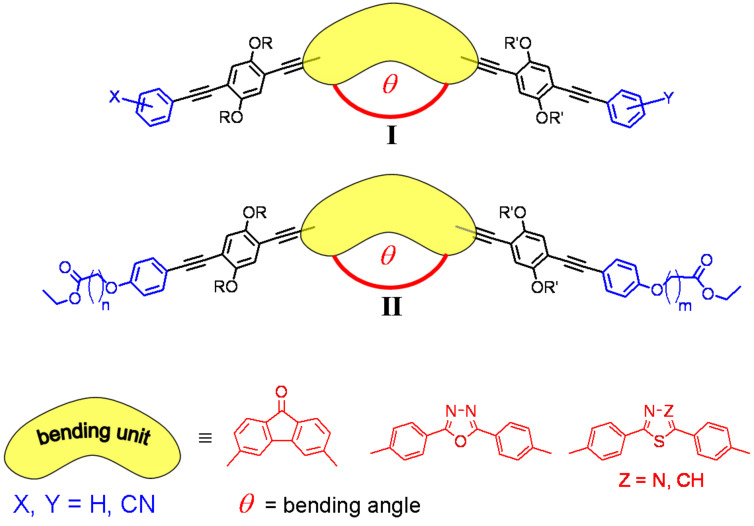
Design of V-shaped, shape-persistent oligo(phenylene ethynylene) mesogens of type **I** and **II** (R, R′ = alkyl chains; n, m = 4–10).

Historically, a biaxial nematic phase was found first in lyotropic mixtures, where the micellar shape can be gradually tuned [6]. A thermotropic biaxial nematic phase of molecules of low molar mass is in high demand because of its potential application in display technology. After the discovery of the biaxial nematic phase in lyotropic materials, many claims of biaxial thermotropic nematic phases were published without being accepted [5]. During this period also banana-shaped molecules were discovered and theoreticians highlighted the possibility to use bent-shaped molecules with rigorously defined shape (bending angle) for the formation of the desired phase [5,7]. But it was not until 2004 that sufficient evidence was presented for biaxiality of nematic phases in the series of V-shaped oxadiazoles by X-ray diffraction and solid-state ^2^H NMR spectroscopy [8–10]. This has been recently confirmed by various other methods [11–14]. Since then several new materials have been designed and reported to be biaxial, among others tetrapodes [15–17] and banana-shaped oligoesters [18–19]. However, there is still a controversial discussion about the phase biaxiality of these materials and their switching behaviour [20–22]. All these latter molecular structures are, however, flexible and can change their conformation and thus their shape. Since theoretically biaxial nematic phases were predicted for a molecule with a defined shape and angle, we aimed to design a shape-persistent molecular scaffold of type **I** (Figure 2) based on oligo(phenylene ethynylene) building blocks. These molecules show liquid crystal behaviour only with a flat bending unit possessing a dipole along the apex of the mesogen, sufficiently long aliphatic chains R and at least one pyridyl or acceptor substituted aromatic unit at the periphery of the molecule [23–24]. Fluorenone [25], oxadiazole [26], thiazole and thiadiazole [24] derivatives have been synthesised and evidence for biaxiality in their monotropic nematic phases has been presented. Monotropic phases are metastable and crystallise, thus making detailed studies of these phases extremely difficult. Therefore, low temperature stable (enantiotropic) phases are urgently demanded. In an earlier theoretical work, weak hydrogen bonds were suggested to possibly induce and stabilise biaxial nematic phases [27]. Therefore, we modified our design concept and attached ester groups to the peripheral aromatic unit via an alkyloxy spacer to obtain the general structures of type **II**. The esters may be subsequently cleaved, in order to generate carboxylic acids and thus hydrogen bonded dimers and oligomers. In this article, the synthesis of a series of thiadiazoles of general structure **II** is presented and the successful approach to low temperature, enantiotropic nematic liquid crystals in the family of bent-shaped oligo(phenylene ethynylenes) will be discussed.

## Results and Discussion

### Synthesis

**Scheme 1:**
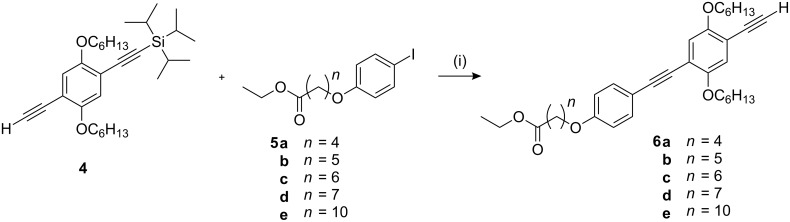
Synthesis of arm derivatives **6**. *Reaction conditions:* (i) 1) Pd(PPh_3_)_4_, CuI, piperidine, rt; 2) TBAF, THF, rt.

**Scheme 2:**
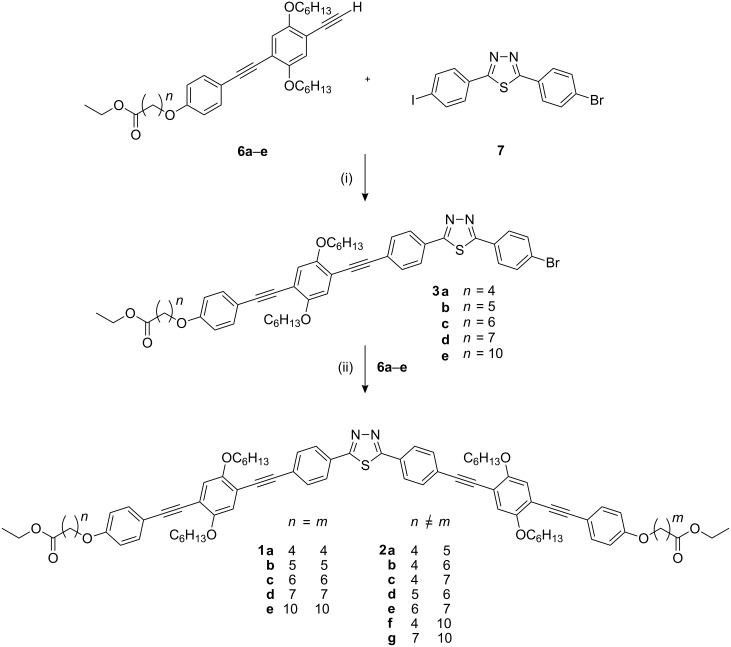
Two-step synthesis of V-shaped nematogens: symmetric (**1**) and non-*C*_2_-symmetric (**2**) thiadiazoles. *Reaction conditions:* (i) Pd(PPh_3_)_4_, CuI, piperidine, rt; (ii) Pd(PPh_3_)_4_, CuI, piperidine, 65 °C.

The shape-persistent arms of the new nematogens were prepared following the recent optimised procedure [25], using the mono-protected diethynylbenzene derivative **4** as a key compound (Scheme 1). The peripheral aromatic units **5** were obtained by etherification of 4-iodophenol with the corresponding ethyl ω-bromoalkanoate [28]. Cross-coupling of iodobenzene **5** with ethynyl compound **4** and subsequent cleavage of the silyl protecting group afforded the arm derivatives **6**. As in the previously published two-step synthesis, the arms **6** were linked successively to the non-symmetric thiadiazole bending unit **7** (Scheme 2) [24]. All compounds were carefully purified and characterised by ^1^H, ^13^C NMR, mass spectrometry and elemental analysis (see experimental section).

### Thermotropic Properties

**Figure 3:**
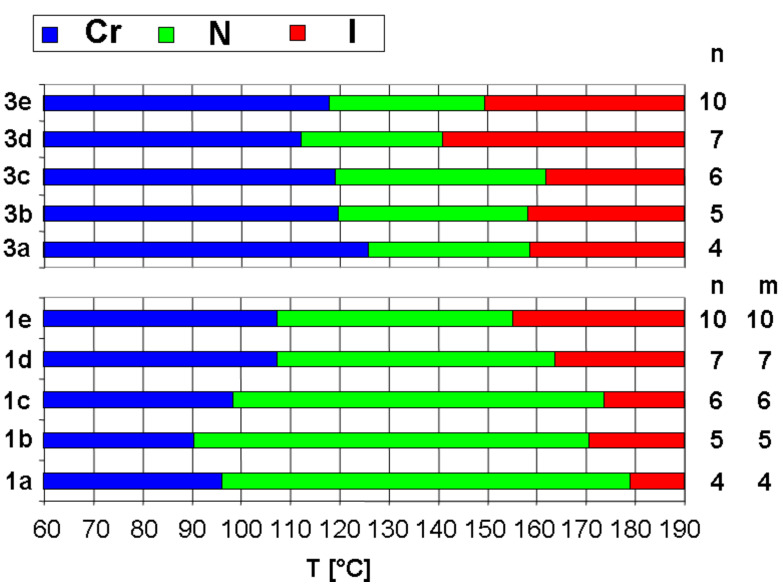
Comparison of the mesophase ranges of intermediate hockey stick compounds **3** and symmetric V-shaped nematogens **1**.

The thermotropic behaviour of all materials was investigated by differential scanning calorimetry (DSC) and polarised optical microscopy (POM). The results are collected in Table 1. Interestingly, all phenylene ethynylene oligomers show exclusively enantiotropic nematic liquid crystal phases, even for the hockey stick shaped intermediates **3**. However, the temperature intervals for the latter are small, approaching a maximum of 43 °C for compound **3c** and melting in all cases occurs only above 100 °C (Figure 3). In this series, an odd-even behaviour becomes apparent for the Cr–N, as well as for the N–I transition with increasing chain length (n = 4–7) [1,29]. It is important to note that for the first three members (**3a**–**c**) there is only a small impact of the chain length on the phase transition temperatures. Only with the heptyl chains do the transition temperatures decrease significantly. A closer look at transition enthalpies and entropies reveal very small values for **3a** and **3b** (Δ*H* = 0.1 kJ·mol^−1^; Δ*S* = 0.2 J·K^−1^·mol^−1^). Entropy values approaching zero, i.e. second order transitions, are predited for direct transitions from the isotropic liquid to the biaxial nematic phase for biaxial molecules [30]. Thus, these hockey stick shaped derivatives may be good candidates for the investigation of the presence of phase biaxiality. POM studies reveal for derivatives **3a**–**e** Schlieren textures with two and four brushed disclinations. Homeotropic alignment to study possible biaxiality of the samples was not obtained. Only for a sample of compound **3a** could planar aligned thin LC films be prepared. Upon rotation of the sample the film became alternately dark at 0° and birefringent at 45°.

**Table 1:** Thermotropic behaviour of hockey stick compounds **3** and V-shaped molecules **1** and **2**.

Compound	Rate 10 °C/min (Onset [°C] / Δ*H* [kJ/mol])^a^	Δ*S*_N_ [J·mol^−1^·K^−1^]

**3a**	**Cr** 126 / *35.1* **N** 159 / *0.1* **I**	0.2
**3b**	**Cr** 120 / *44.0* **N** 158 / *0.1* **I**	0.2
**3c**	**Cr** 119 / *47.0* **N** 162 / 0.9 **I**	2.1
**3d**	**Cr** 112 / 42.6 **N** 141 / 1.4 **I**	3.4
**3e**	**Cr** 118 / *58.0* **N** 150 / *0.9* **I**	2.1
**1a**	**Cr** 96 / *65.9* **N** 179 / *1.8* **I**	4.0
**1b**	**Cr** 91 / *55.6* **N** 171 / *1.6* **I**	3.6
**1c**	**Cr** 99 / *56.5* **N** 174 / *1.8* **I**	4.0
**1d**	**Cr** 108 / *57.6* **N** 164 / *1.9* **I**	4.4
**1e**	**Cr** 108 / *117.7* **N** 155 / *1.8* **I**	4.2
**2a**	**Cr** 88 / *102.3* **N** 179 / *1.9* **I**	4.2
**2b**	**Cr** 68 / *38.5*^b^ **N** 178 / *1.9* **I**	4.2
**2c**	**Cr** 67 / *37.0*^b^ **N** 175 / *1.8* **I**	4.0
**2d**	**Cr** 89 / *48.3* **N** 175 / *1.7* **I**	3.8
**2e**	**Cr** 96 / *55.5* **N** 171 / *1.8* **I**	4.1
**2f**	**Cr** 70 / *37.3*^b^ **N** 168 / *1.7* **I**	3.9
**2g**	**Cr** 77 / *53.6*^b^ **N** 162 / *1.7* **I**	3.9

^a^Data are given for the second heating. ^b^Data for the first heating.

**Figure 4:**
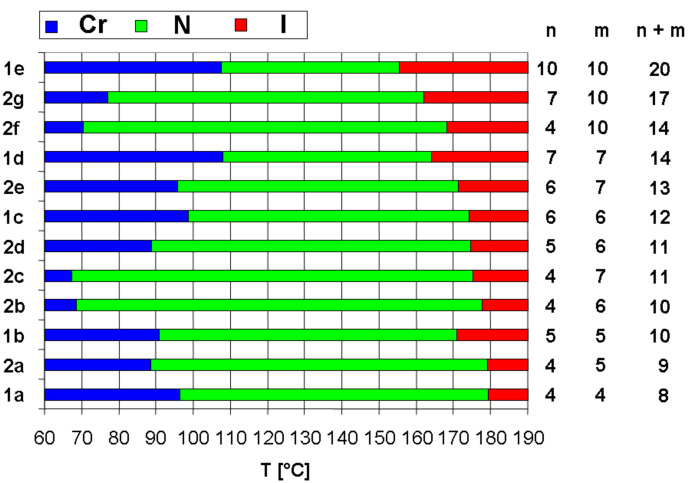
Comparison of the thermal behaviour of symmetric and non-symmetric V-shaped molecules. The molecules are ordered from the bottom to the top by increasing total number of spacer CH_2_ groups (*n* + *m*).

Figure 3 compares the transitions of symmetric V-shaped compounds **1a**–**e**. The transition temperatures I–N decrease from compounds with a short peripheral spacer between the aromatic and the ester group to long spacer derivatives. A clear odd-even effect is revealed. The transition enthalpies and entropies are relatively high (Δ*H* = 1.6–1.9 kJ·mol^−1^; Δ*S* = 3.6–4.2 J·K^−1^·mol^−1^) pointing to first order transitions. Melting temperatures and melting enthalpies follow a different progression; they decrease from **1a** to **1b** and increase again from **1b** to **1e**. All melting temperatures are relatively high (above 90 °C). In order to lower the latter, non-symmetric V-shaped mesogens **2** with two arms consisting of different peripheral building blocks have been prepared. The series of molecules **2a**–**c**, **2f** with a pentanoic acid ethyl ester group on one side shows a decrease in melting and clearing temperatures with increasing spacer lengths on the other arm. The decrease of melting temperature dominates and reaches a minimum for molecule **2c** with an octanoic acid ethyl ester as a peripheral group. In this series of molecules no apparent odd-even effect can be monitored. The thermotropic properties in comparison with the increasing lengths of the peripheral alkanoic acid ethyl ester spacers are illustrated in Figure 4. Apparently, the clearing temperature decreases with the total number of peripheral methylene groups (from 179.1 °C for **1a** to 155.2 °C for **1e**). Note that non-symmetric compounds always possess lower melting and higher clearing temperatures compared to their symmetric counterparts with the same number of methylene groups (compare **1b**/**2b** and **1d**/**2f**). It appears that a large difference in chain lengths results in higher stability of the mesophase, i.e. a low crystallisation tendency (see **2f**, **2g**, **2b** and **2c** and compare to **2d** and **2a**). Maximum LC temperature intervals for enantiotropic liquid crystalline phases of 109 °C and 108 °C were found for **2b** and **2c**, showing the success of the strategy for this series of compounds. Note, as indicated in Table 1, that some of the samples can be supercooled without crystallisation. Some materials can be stored for more than 1 h at 25 °C without visible formation of crystal grains.

**Figure 5:**
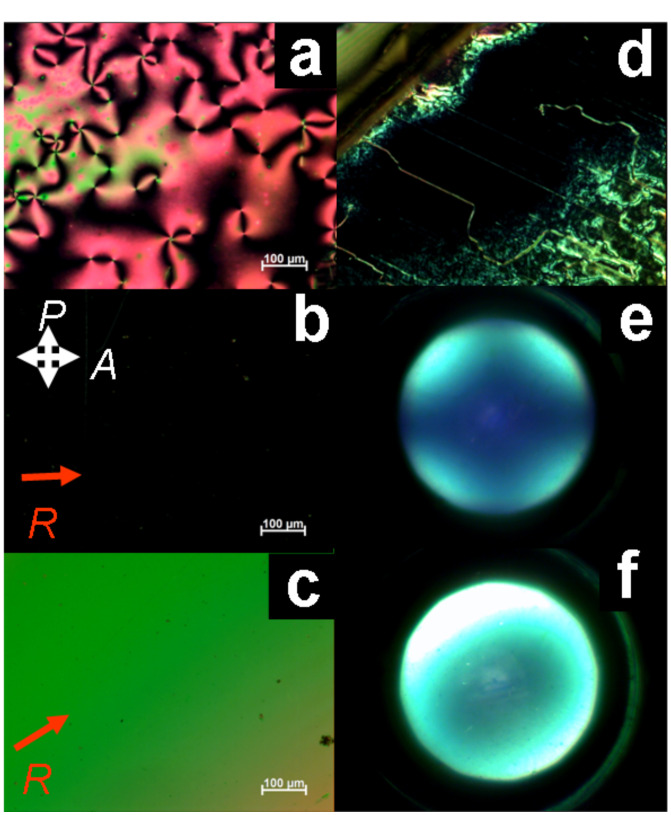
Textures of the nematic phase of **2c**. a) Schlieren texture at 173 °C. b) and c) Planar alignment on rubbed polyimide at 167 °C (R = rubbing direction). d) Homeotropic aligned area of **2c** on glass at 163 °C. e) Conoscopic picture at 161 °C and f) Conoscopic picture using the circular polariser.

Microscopy studies were performed to examine the nature of the mesophases. POM revealed for all samples Schlieren textures with two and four brushed disclinations (Figure 5) typically observed for nematic phases. The high mobility of the phases, combined with a blaze of colours upon external pressure and the absence of homeotropic alignment after shearing, is also a sign of their nematic nature. The nematic materials aligned preferentially planar on conventional glass substrates and between glass coated with antiparallel rubbed polyimide alignment layers. Rotation of the samples exhibited alternately birefringent and dark textures. Conoscopy switched between a blurry conoscopic cross and birefringent photographs. However, the circular polariser could not reveal any optical axes. These results would be expected for uniaxial as well as biaxial nematic phases with planar alignment. Only homeotropic aligned samples allow distinction between uniaxial and biaxial phases. In the case of **2c** small homeotropically aligned areas could be obtained on glass substrates. Conoscopy revealed positive optical anisotropy, thus the molecules’ long axes are aligned perpendicular to the glass substrate. The black texture between the crossed polarisers at all rotation angles of the sample indicates the uniaxial nature right after the phase transition, further confirmed by the conoscopic cross. Upon cooling, the sample becomes slightly birefringent in some areas. This process yielded an inhomogenous texture pointing to the formation of multiple small domains. The domains which remained dark revealed a conoscopic cross at all temperatures. The symmetry of the cross changed only slightly upon rotation of the sample. These observations are in good agreement with the uniaxial nature of the nematic phase of nematogen **2c** even at room temperature.

### X-ray Diffraction

**Figure 6:**
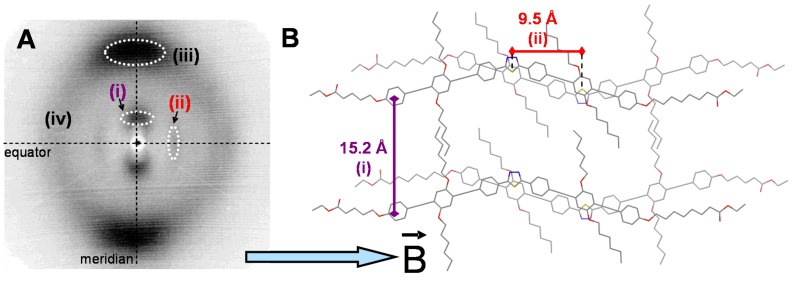
X-ray study of nematic mesophases from V-shaped mesogens. A: Diffraction pattern of **2c** at 70 °C. B: Model of the local molecular order and characteristic distances corresponding to reflections (i) and (ii).

X-ray studies were performed on magnetic-field-aligned samples with the X-ray beam perpendicular to the alignment direction. Figure 6A shows a typical X-ray pattern found for all investigated derivatives with three diffuse pairs of reflections (i–iii) and a halo (iv) corresponding to the average separation of the liquid-like chains. As shown in Figure 6B, reflections (i) are assigned to the separation of the molecules along the bisector. Reflections (ii) correspond to a distance which can be rationalised by the separation of two antiparallel thiadiazole rings along the molecular long axis. Reflections (iii) are typical for the π–π distance between conjugated molecules. Note that for reflections (i) and (iii) the reflection conditions cannot be simultaneously fulfilled. This points to the fact that at least two distinct domains are observed by the experiment [31–32].

Table 2 summarises the Bragg distances *d* together with the correlation lengths ξ/*d* obtained by the Scherrer formula [33–34]. It reveals that the values *d*(i–iv) are not a function of the spacer lengths between peripheral aromatic units and the ester groups. For example *d*(i) distances are almost constant in the range of 15–16 Å although the molecular length increases by 12 CH_2_ units from compound **1a** to **1e**. Even the distances *d*(ii), attributed to the molecular long axis, on which the different spacer length should have the largest impact, remain constant between 9 and 10 Å. The latter can be rationalised when the electron-poor thiadiazole units interact with the electron-rich 2,5-dialkyloxybenzenes of an antiparallel aligned mesogen. As illustrated in Figure 6B, the sulfur atoms are then separated throughout the sample on average by 9–10 Å. The fact that with small angle X-ray scattering no reflection corresponding to the overall molecular length could be found is not fully understood. The correlation lengths ξ/*d* are all in the range of 3–5 repeating units indicating the absence of any long range positional order and thus confirming the nematic nature of the mesophases. The π–π-distances are relatively large and only marginally smaller than the average separation of alkyl chains. However, at this temperature range similar values have been obtained previously in the series of fluorenone derivatives, in which the π–π interaction increased with decreasing temperature and moderated the uniaxial to biaxial transition [25].

**Table 2:** X-ray diffraction data. The correlation length ξ was estimated from the half width of the reflections by using the Scherrer formula [33–34].

Compound	T [°C]	*d* (i) [Å](ξ/*d*)	*d* (ii) [Å](ξ/*d*)	*d* (iii) [Å](ξ/*d*)	*d* (iv) [Å](ξ/*d*)

**1a**	105	15.1 (4.0)	10.2 (4.2)	4.3 (5.2)	4.6 (4.3)
**2a**	80	14.9 (4.1)	9.8 (4.4)	4.2 (5.3)	4.4 (4.7)
**1c**	100	15.7 (3.4)	9.3 (4.3)	4.4 (5.4)	4.6 (4.1)
**2c**	70	15.2 (2.4)	9.5 (6.3)	4.0 (6.1)	4.7 (3.7)
**1e**	105	16.4 (2.8)	9.5 (4.3)	4.4 (5.4)	4.6 (3.9)

X-ray diffraction shows the orientation of two molecular axes in this mesogen family, which may be an indication for phase biaxiality. In contrast, the optical experiments of compound **2c** point to the uniaxial nature of its nematic phase. In order to rationalise these two different results, it can be assumed on the bases of the correlation lengths that the thiadiazoles form small aggregates. These aggregates are responsible for the observed diffuse X-ray pattern, however, they either rotate about their long axis or the two different axes of the aggregates are isotropically distributed around the direction of the magnetic field, eventually resulting in a uniaxial phase even at room temperature. The latter model has been recently suggested by a theoretical work from Vanakaras [35], in which three different uniaxial and biaxial nematic phases based on aggregates or clusters have been proposed. The model is further supported by results obtained from a bent-shaped oxazole derivative, which forms polar clusters in the nematic phase [36]. However, further work is in progress in order to draw a more detailed picture of the supramolecular organisation of thiadiazole derivatives **1** and **2** in their nematic phase.

## Conclusion

Thiadiazole nematogens with ester groups connected via alkyloxy spacers could be efficiently prepared by a previously reported two-step procedure. These mesogens are capable of hydrogen bonding if the esters are cleaved. The mesophase range and the melting temperature reach an optimum when the nematogens are desymmetrised with a butoxy and a heptyloxy spacer. For this molecule, optical observations by conoscopy monitored a uniaxial nematic phase over the whole temperature range of 150 °C upon cooling from the isotropic phase to room temperature. X-ray diffraction points to the alignment of two axes in the magnetic field in this family of mesogens. Both features may be rationalised if larger assemblies of V-shaped molecules are isotropically distributed around the direction of the magnetic field, thus leading only to uniaxial nematic phases. Work is in progress to synthesise molecules with a smaller bending angle in order to induce possibly a biaxial order at high temperature.

## Experimental

Chemicals were obtained from Fisher Scientific and Sigma-Aldrich and used as received. The synthesis of compounds **4** [23,25] and **7** [24] was described previously. Column chromatography was carried out on silica 60 (Merck, mesh 70–230). PFT ^1^H and ^13^C NMR spectra were recorded in CDCl_3_ with a Varian Oxford 400 MHz spectrometer with the residual solvent signal at 7.26 ppm as a reference. Mass spectra were obtained on a Finnigan MAT95 (FD MS). Elemental analysis was carried out in the microanalytical laboratory at the University of Mainz. POM observations were made with a Zeiss Axioscop 40 equipped with a Linkam THMS600 hot stage. DSC was performed using a Perkin Elmer Pyris 1.

X-ray diffraction measurements were carried out on powder samples in glass capillaries of 1.5 mm diameter. The nematic phases were aligned in a magnetic field (1T) upon cooling from the isotropic to the nematic phase. The WAXS measurements were performed by using a standard copper anode (2.2 kW) source with pinhole collimation equipped with a X-ray mirror (Osmic typ CMF15-sCu6) and a Bruker detector (High-star) with 1024 × 1024 pixels. The diffraction data were calibrated by using silver behenate as a calibration standard [37]. The X-ray patterns were evaluated using the datasqueeze software (http://www.datasqueezesoftware.com/).

### General method for preparation of intermediate products **3a**–**e**

The mixture of 1.0 equiv of thiadiazole **7**, 1.0 equiv of the corresponding terminal alkyne **6a**–**e**, 0.1 equiv of Pd(PPh_3_)_4_ and 0.05 equiv of CuI in piperidine is stirred for 2 h at room temperature. Subsequently, the solvent is removed *in vacuo* and the products are isolated by column chromatography using a mixture of EtOAc/hexane.

**2-{4-[4-{4-[4-(Ethoxycarbonyl)butoxy]phenylethynyl}-2,5-bis(hexyloxy)phenyl]ethynylphenyl}-5-(4-bromophenyl)-1,3,4-thiadiazole (3a**) Hexane/EtOAc = 6/1 (*R_f_* = 0.25), yellow solid; yield 0.12 g (78%). ^1^H NMR (400 MHz, CDCl_3_): δ = 7.99 (2H, AA′BB′), 7.89 (2H, AA′BB′), 7.64 (4H, AA′BB′), 7.46 (2H, AA′BB′), 7.02 (s, 1H), 7.01 (s, 1H), 6.86 (2H, AA′BB′), 4.14 (q, 2H, COOCH_2_CH_3_, *J* = 7.2), 4.05 (t, 2H, OCH_2_, *J* = 6.4), 4.04 (t, 2H, OCH_2_, *J* = 6.4), 3.99 (m, 2H, OCH_2_), 2.39 (t, 2H, CH_2_COOEt, *J* = 7.2), 1.85 (m, 8H, CH_2_); 1.55 (m, 4H, CH_2_); 1.36 (m, 8H, CH_2_); 1.26 (t, 3H, CH_3_, *J* = 7.2); 0.91 (t, 3H, CH_3_, *J* = 7.2), 0.90 (t, 3H, CH_3_, *J* = 7.2); ^13^C NMR (100 MHz, CDCl_3_): δ = 173.8 (C_q_, C=O), 167.9, 167.2 (C_q_, N=C–S); 159.3 (C_q_, C–OCH_2_), 154.0, 153.3 (C_q_, C–OC_6_H_13_), 133.2, 132.6, 132.4 (C_t_), 129.4 (C_t_), 129.1 (C_q_), 127.9 (C_t_), 126.8, 125.8 (C_q_), 117.0, 116.8 (C_t_); 115.4, 115.2 (C_q_), 114.6 (C_t_), 112.9 (C_q_), 95.6, 94.0, 89.2, 84.7 (C≡C), 69.8, 69.7, 67.8 (OCH_2_), 60.4 (COOCH_2_CH_3_), 34.4 (CH_2_COOEt), 31.7, 29.5, 29.4, 29.0, 25.9, 25.8, 24.8, 22.8 (CH_2_), 14.4, 14.2 (CH_3_); EA: Calc. for C_59_H_53_BrN_2_O_5_S: C 68.28, H 6.20, N 3.25, S 3.72; Found: C 68.38, H 6.31, N 3.33, S 3.70; FD MS: *m/z* [%]:861.7 (87, [M+2]^+^); 859.7 (100, M^+^).

### General method for preparation of V-shaped molecules **1a**–**e** and **2a**–**g**

The mixture of 1.0 equiv of thiadiazole derivatives **3a**–**e**, 1.0 equiv of the corresponding terminal alkine **6a**–**e**, 0.2 equiv of Pd(PPh_3_)_4_ and 0.1 equiv of CuI in piperidine is stirred for 2 h at 65 °C. The solvent is then removed *in vacuo* and the products are isolated by means of column chromatography using a mixture of EtOAc/hexane.

**2,5-Bis-{4-[4-{4-[4-(ethoxycarbonyl)butoxy]phenylethynyl}-2,5-bis(hexyloxy)phenyl]ethynylphenyl}-1,3,4-thiadiazole (1a)** Hexane/EtOAc = 6/1 (*R_f_* = 0.1), yellow solid; yield 120 mg (74%). ^1^H NMR (400 MHz, CDCl_3_): δ = 8.00 (4H, AA′BB′), 7.64 (4H, AA′BB′), 7.46 (4H, AA′BB′), 7.02 (s, 2H), 7.01 (s, 2H), 6.86 (4H, AA′BB′), 4.13 (q, 4H, COOCH_2_CH_3_, *J* = 7.2), 4.04 (t, 4H, OCH_2_, *J* = 6.4), 4.03 (t, 4H, OCH_2_, *J* = 6.4), 3.98 (t, 4H, OCH_2_, *J* = 6.4), 2.34 (t, 4H, CH_2_COOEt, *J* = 7.2), 1.85 (m, 16H, CH_2_), 1.55 (m, 8H, CH_2_), 1.37 (m, 16H, CH_2_), 1.26 (t, 6H, CH_3_, *J* = 7.2), 0.91 (t, 6H, CH_3_, *J* = 7.2), 0.90 (t, 6H, CH_3_, *J* = 7.2); ^13^C NMR (100 MHz, CDCl_3_): δ = 173.6 (C_q_, C=O), 167.8 (C_q_, N=C–S), 159.2 (C_q_, C–OCH_2_), 154.0, 153.5 (C_q_, C–OC_6_H_13_), 133.2, 132.3 (C_t_), 129.6 (C_q_), 127.9 (C_t_), 126.7 (C_q_), 117.0, 116.8 (C_t_), 115.5, 115.2 (C_q_), 114.6 (C_t_), 112.9 (C_q_), 95.5, 94.1, 89.2, 84.7 (C≡C), 69.8, 69.7, 67.6 (OCH_2_), 60.5 (COOCH_2_CH_3_), 34.1 (CH_2_COOEt), 31.8, 29.4, 28.7, 25.9, 22.81, 22.79, 21.7 (CH_2_), 14.4, 14.2 (CH_3_); EA: Calc. for C_84_H_98_N_2_O_10_S: C 75.99, H 7.44, N 2.11, S 2.41; Found: C 75.94, H 7.47, N 2.05, S 2.42; FD MS: *m/z* [%]: 1325.9 (100, M^+^); 663.1 (70, M^2+^).

## Supporting Information

File 1 Synthetic procedures and analytical results for compounds **1b**–**e**, **2a**–**g**, **3b**–**e**, **5a**–**e** and **6a**–**e**.
