# Supplementary material for: Low temperature enantiotropic nematic phases from V-shaped, shape-persistent molecules
Source: Beilstein J Org Chem. 2009 Dec 4;5:73. doi: 10.3762/bjoc.5.73 (PMC2839554; doi:10.3762/bjoc.5.73)
Supplement: File 1 — Synthetic procedures and analytical results for compounds 1b–e, 2a–g, 3b–e, 5a–e and 6a–e. [file Beilstein_J_Org_Chem-05-73-s001.doc]

# Supporting Information

**Low temperature enantiotropic nematic phases from V-shaped, shape-persistent molecules**

Matthias Lehmann^*^ and Jens Seltmann

Address: Institute of Chemistry, Chemnitz University of Technology, Straße der Nationen 62, 09111 Chemnitz, Germany

Email: Matthias Lehmann - [Matthias.Lehmann@chemie.tu-chemnitz.de](mailto:Matthias.Lehmann@chemie.tu-chemnitz.de)

^*^Corresponding author

**General procedure for preparation of iodobenzenes 5a–e**

In a nitrogen atmosphere a mixture of 1.0 equiv of 4-iodophenol, 1.3 equiv of K_2_CO_3_ in dried DMF was heated to 55 °C. Thereto 1.0 equiv of ethyl *ω*-bromoalkanoate was added and the mixture was stirred over night at this temperature. Water and ethyl acetate were added and the product was extracted into the organic solvent. The organic solution was washed with saturated brine and dried with anhydrous sodium sulphate. The extract was filtered and concentrated *in vacuo*.

**Ethyl 5-(4-iodophenoxy)pentanoate (5a)** bright purple oil; yield 2.9 g (92%). ^1^H NMR (400 MHz, CDCl_3_): δ = 7.53 (2H, AA’BB’), 6.65 (2H, AA’BB’), 4.12 (q, 2H, COOCH_2_CH_3_, *J* = 7.2), 3.92 (m, 2H, OCH_2_), 2.36 (m, 2H, CH_2_–COOEt), 1.80 (m, 4H, CH_2_), 1.25 (t, 3H, CH_3_, *J* = 7.2); ^13^C NMR (100 MHz, CDCl_3_): δ = 173.3 (C_q_, C=O), 158.7 (C_q_, C–OCH_2_), 138.1 (C_t_), 116.8 (C_t_), 82.6 (C_q_, C–I), 67.4 (OCH_2_), 60.3 (COOCH_2_CH_3_), 33.8 (CH_2_COOEt), 28.5, 21.5 (CH_2_), 14.3 (CH_3_).

**Ethyl 6-(4-iodophenoxy)hexanoate (5b)** bright purple oil; yield 2.9 g (92%). NMR data are identical to whose reported in literature [1].

**Ethyl 7-(4-iodophenoxy)heptanoate (5c)** bright orange oil; yield 2.9 g (84%). ^1^H NMR (400 MHz, CDCl_3_): δ = 7.51 (2H, AA’BB’), 6.64 (2H, AA’BB’), 4.11 (q, 2H, OCH_2_CH_3_, *J* = 7.2), 3.88 (t, 2H, OCH_2_, *J* = 6.8), 2.29 (t, 2H, CH_2_COOEt, *J* = 7.4), 1.75 (m, 2H, CH_2_), 1.64 (m, 2H, CH_2_), 1.47–1.35 (m, 4H, CH_2_), 1.24 (t, 3H, CH_3_, *J* = 7.1); ^13^C NMR (100 MHz, CDCl_3_): δ = 173.8 (C_q_, C=O), 159.0 (C_q_, C–OCH_2_), 138.2 (C_t_), 116.9 (C_t_), 82.5 (C_q_, C–I), 67.9 (OCH_2_), 60.3 (COOCH_2_CH_3_), 34.3 (CH_2_COOEt), 29.0, 28.9, 25.7, 24.9 (CH_2_), 14.3 (CH_3_).

**Ethyl 8-(4-iodophenoxy)octanoate (5d)** bright purple oil; yield 2.3 g (86%). ^1^H NMR (400 MHz, CDCl_3_): δ = 7.51 (2H, AA’BB’), 6.63 (2H, AA’BB’), 4.10 (q, 2H, OCH_2_CH_3_, *J* = 7.2), 3.87 (t, 2H, OCH_2_, *J* = 6.4), 2.28 (t, 2H, CH_2_COOEt, *J* = 7.2), 1.73 (m, 2H, CH_2_), 1.61 (m, 2H, CH_2_), 1.47–1.32 (m, 6H, CH_2_), 1.24 (t, 3H, CH_3_, *J* = 7.2); ^13^C NMR (100 MHz, CDCl_3_): δ = 173.9 (C_q_, C=O), 159.0 (C_q_, C–OCH_2_), 138.2 (C_t_), 116.9 (C_t_), 82.5 (C_q_, C–I), 67.9 (OCH_2_), 60.3 (COOCH_2_CH_3_), 34.3 (CH_2_COOEt), 29.1, 29.0, 25.8, 24.9 (CH_2_), 14.3 (CH_3_).

**Ethyl 11-(4-iodophenoxy)undecanoate (5e)** fawn solid; yield 2.6 g (88%). ^1^H NMR (400 MHz, CDCl_3_): δ = 7.52 (2H, AA’BB’), 6.65 (2H, AA’BB’), 4.12 (q, 2H, OCH_2_CH_3_, *J* = 7.2), 3.90 (t, 2H, OCH_2_, *J* = 6.4), 2.28 (t, 2H, CH_2_COOEt, *J* = 7.2), 1.75 (m, 2H, CH_2_), 1.61 (m, 2H, CH_2_), 1.45–1.26 (m, 10H, CH_2_), 1.25 (t, 3H, CH_3_, *J* = 7.2); ^13^C NMR (100 MHz, CDCl_3_): δ = 174.1 (C_q_, C=O), 159.1 (C_q_, C–OCH_2_), 138.2 (C_t_), 117.0 (C_t_), 82.5 (C_q_, C–I), 68.2 (OCH_2_), 60.3 (COOCH_2_CH_3_), 34.5 (CH_2_COOEt), 29.6, 29.48, 29.44, 29.36, 29.2, 26.1, 25.1 (CH_2_), 14.4 (CH_3_).

**General procedure for preparation of terminal alkynes 6a–e**

A mixture of 1.0 equiv of alkyne **4**, 1.0 equiv of the corresponding iodophenol derivative **5a**–**e**, 0.1 equiv of Pd(PPh_3_)_4_ and 0.05 equiv of CuI in 50 ml piperidine is stirred for 1 h at room temperature. Afterwards the solvent is removed *in vacuo* and the intermediate products are isolated by column chromatography using a mixture of EtOAc/hexane. These products are treated with 1.5 equiv of TBAF in 40 ml THF and are stirred for 1 h at room temperature. After removing the solvent *in vacuo* the terminal alkynes **6a**–**e** are purified by column chromatography with a mixture of EtOAc/hexane.

**Ethyl 5-[4-({2,5-bis(hexyloxy)-4-(ethynyl)phenyl}ethynyl)phenoxy]pentanoate (6a)** Hexane/EtOAc = 5/1 (*R_f_* = 0.45), bright yellow solid; yield 0.38 g (80%). ^1^H NMR (400 MHz, CDCl_3_): δ = 7.45 (2H, AA’BB’), 6.97 (s, 1H), 6.96 (s, 1H), 6.85 (2H, AA’BB’), 4.11 (q, 2H, COOCH_2_CH_3_, *J* = 7.2), 3.98 (m, 6H, OCH_2_), 3.33 (s, 1H, C≡CH), 2.34 (m, 2H, CH_2_COOEt), 1.83 (m, 8H, CH_2_), 1.52 (m, 4H, CH_2_), 1.35 (m, 8H, CH_2_), 1.22 (t, 3H, CH_3_, *J* = 6.8), 0.90 (m, 6H, CH_3_); ^13^C NMR (100 MHz, CDCl_3_): δ = 173.6 (C_q_, C=O), 158.1 (C_q_, C–OCH_2_), 154.3, 153.4 (C_q_, C–OC_6_H_13_), 133.3 (C_t_), 117.9, 116.8 (C_t_), 115.5, 115.2 (C_q_), 114.6 (C_t_), 112.1 (C_q_), 95.2, 84.5, 82.2, 80.2 (C≡C), 69.8, 69.7, 67.6 (OCH_2_), 60.5 (COOCH_2_CH_3_), 34.1 (CH_2_COOEt), 31.74, 31.67, 29.4, 29.2, 28.7, 25.9, 25.7, 22.8, 22.7, 21.8 (CH_2_), 14.4, 14.2 (CH_3_); EA: Calc. for C_35_H_46_O_5_: C 76.89, H 8.48; Found: C 76.82, H 8.50; FD MS: *m/z* [%]: 548.4 (7, [M+2]^+^); 547.4 (41, [M+1]^+^); 546.4 (100, M^+^).

**Ethyl 6-[4-({2,5-bis(hexyloxy)-4-(ethynyl)phenyl}ethynyl)phenoxy]hexanoate (6b)** Hexane/EtOAc = 5/1 (*R_f_* = 0.4), bright yellow solid; yield 0.5 g (58%). ^1^H NMR (400 MHz, CDCl_3_): δ = 7.45 (2H, AA’BB’), 6.97 (s, 1H), 6.96 (s, 1H), 6.85 (2H, AA’BB’), 4.13 (q, 2H, COOCH_2_CH_3_, *J* = 7.2), 3.98 (m, 6H, OCH_2_), 3.33 (s, 1H, C≡CH), 2.34 (t, 2H, CH_2_COOEt, *J* = 7.2), 1.81 (m, 8H, CH_2_), 1.71 (m, 2H, CH_2_), 1.52 (m, 4H, CH_2_), 1.34 (m, 8H, CH_2_), 1.26 (t, 3H, CH_3_, *J* = 7.2), 0.90 (m, 6H, CH_3_); ^13^C NMR (100 MHz, CDCl_3_): δ = 173.8 (C_q_, C=O), 159.3 (C_q_, C–OCH_2_), 154.3, 153.3 (C_q_, C–OC_6_H_13_), 133.3 (C_t_), 117.9, 116.8 (C_t_), 115.4, 115.2 (C_q_), 114.5 (C_t_), 112.1 (C_q_), 95.2, 84.4, 82.2, 80.2 (C≡C), 69.71, 69.67, 67.8 (OCH_2_), 60.4 (COOCH_2_CH_3_), 34.3 (CH_2_COOEt), 31.7, 31.6, 29.2, 29.2, 29.0, 25.8, 25.7, 24.8, 22.74, 22.69 (CH_2_), 14.3, 14.2, 14.1 (CH_3_); EA: Calc. for C_36_H_48_O_5_: C 77.11, H 8.63; Found: C 76.87, H 8.76; FD MS: *m/z* [%]: 559.9 (100, M^+^).

**Ethyl 7-[4-({2,5-bis(hexyloxy)-4-(ethynyl)phenyl}ethynyl)phenoxy]heptanoate (6c)** Hexane/EtOAc = 10/1 (*R_f_* = 0.4), bright yellow solid; yield 0.75 g (70%). ^1^H NMR (400 MHz, CDCl_3_): δ = 7.45 (2H, AA’BB’), 6.97 (s, 1H), 6.96 (s, 1H), 6.85 (2H, AA’BB’), 4.13 (q, 2H, COOCH_2_CH_3_, *J* = 7.2), 3.98 (m, 6H, OCH_2_), 3.33 (s, 1H, C≡CH), 2.31 (t, 2H, CH_2_COOEt, *J* = 7.2), 1.81 (m, 6H, CH_2_), 1.66 (m, 2H, CH_2_), 1.53–1.32 (m, 16H, CH_2_), 1.25 (t, 3H, CH_3_, *J* = 7.2), 0.90 (m, 6H, CH_3_); ^13^C NMR (100 MHz, CDCl_3_): δ = 173.9 (C_q_, C=O), 159.4 (C_q_, C–OCH_2_), 154.3, 153.4 (C_q_, C–OC_6_H_13_), 133.2 (C_t_), 117.9, 116.8 (C_t_), 115.4, 115.2 (C_q_), 114.6 (C_t_), 112.1 (C_q_), 95.3, 84.4, 82.2, 80.2 (C≡C), 69.75, 67.70 (OCH_2_), 60.4 (COOCH_2_CH_3_), 34.4 (CH_2_COOEt), 31.7, 31.6, 29.4, 29.2, 29.1, 29.0, 25.8, 25.7, 24.9, 22.8, 22.7 (CH_2_), 14.4, 14.2 (CH_3_); EA: Calc. for C_37_H_50_O_5_: C 77.31, H 8.77; Found: C 77.09, H 8.88; FD MS: *m/z* [%]: 574.0 (100, M^+^).

**Ethyl 8-[4-({2,5-bis(hexyloxy)-4-(ethynyl)phenyl}ethynyl)phenoxy]octanoate (6d)** Hexane/EtOAc = 15/1 (*R_f_* = 0.25), colourless solid; yield 0.64 g (90%). ^1^H NMR (400 MHz, CDCl_3_): δ = 7.45 (2H, AA’BB’), 6.97 (s, 1H), 6.96 (s, 1H), 6.85 (2H, AA’BB’), 4.11 (q, 2H, COOCH_2_CH_3_, *J* = 7.2), 3.98 (m, 6H, OCH_2_), 3.33 (s, 1H, C≡CH), 2.30 (t, 2H, CH_2_–COOEt, *J* = 7.2), 1.80 (m, 6H, CH_2_), 1.64 (m, 2H, CH_2_), 1.48 (m, 6H, CH_2_), 1.35 (m, 12H, CH_2_), 1.25 (t, 3H, CH_3_, *J* = 7.2), 0.90 (m, 6H, CH_3_); ^13^C NMR (100 MHz, CDCl_3_): δ = 174.0 (C_q_, C=O), 159.4 (C_q_, C–OCH_2_), 154.3, 153.3 (C_q_, C–OC_6_H_13_), 133.2 (C_t_), 117.9, 116.8 (C_t_), 115.3, 115.2 (C_q_), 114.6 (C_t_), 112.1 (C_q_), 95.3, 84.4, 82.2, 80.2 (C≡C), 69.8, 69.7, 68.1 (OCH_2_), 60.3 (COOCH_2_CH_3_), 34.5 (CH_2_COOEt), 31.74, 31.67, 29.4, 29.2, 29.17, 29.15, 26.0, 25.9, 25.7, 25.0, 22.8, 22.7 (CH_2_), 14.4, 14.20, 14.18 (CH_3_); FD MS: *m/z* [%]: 588.8 (45, [M+1]^+^); 587.8 (100, M^+^).

**Ethyl 11-[4-({2,5-Bis(hexyloxy)-4-(ethynyl)phenyl}ethynyl)phenoxy]undecanoate (6e)** Hexane/EtOAc = 15/1 (*R_f_* = 0.25), colourless solid; yield 1.2 g (87%). ^1^H NMR (400 MHz, CDCl_3_): δ = 7.45 (2H, AA’BB’), 6.97 (s, 1H), 6.96 (s, 1H), 6.85 (2H, AA’BB’), 4.13 (q, 2H, COOCH_2_CH_3_, *J* = 7.2), 3.98 (m, 6H, OCH_2_), 3.33 (s, 1H, C≡CH), 2.28 (t, 2H, CH_2_–COOEt, *J* = 7.2), 1.86–1.72 (m, 6H, CH_2_), 1.61 (m, 2H, CH_2_), 1.56–1.40 (m, 6H, CH_2_), 1.39–1.26 (m, 18H, CH_2_), 1.25 (t, 3H, CH_3_, *J* = 7.2), 0.89 (m, 6H, CH_3_); ^13^C NMR (100 MHz, CDCl_3_): δ = 174.0 (C_q_, C=O), 159.4 (C_q_, C–OCH_2_), 154.3, 153.3 (C_q_, C–OC_6_H_13_), 133.1 (C_t_), 117.9, 116.7 (C_t_), 115.3, 115.2 (C_q_), 114.6 (C_t_), 112.1 (C_q_), 95.3, 84.4, 82.2, 80.2 (C≡C), 69.72, 69.67, 68.1 (OCH_2_), 60.3 (COOCH_2_CH_3_), 34.5 (CH_2_COOEt), 31.73, 31.65, 29.6, 29.48, 29.47, 29.41, 29.36, 29.30, 29.24, 26.1, 25.8, 25.7, 25.1, 22.8, 22.7 (CH_2_), 14.4, 14.17, 14.16 (CH_3_).

Analytical data of hockey stick compounds **3b–e**.

**2-{4-[4-{4-[(5-Ethoxycarbonyl)pentyloxy]phenylethynyl}-2,5-bis(hexyloxy)phenyl]­ethynylphenyl}-5-(4-bromophenyl)-1,3,4-thiadiazole (3b)** Hexane/EtOAc = 6/1 (*R_f_* = 0.25), yellow solid; yield 0.15 g (57%). ^1^H NMR (400 MHz, CDCl_3_): δ = 7.99 (2H, AA’BB’), 7.88 (2H, AA’BB’), 7.64 (4H, AA’BB’), 7.45 (2H, AA’BB’), 7.02 (s, 1H), 7.01 (s, 1H), 6.85 (2H, AA’BB’), 4.13 (q, 2H, COOCH_2_CH_3_, *J* = 7.2), 4.04 (t, 2H, OCH_2_, *J* = 6.4), 4.03 (t, 2H, OCH_2_, *J* = 6.4), 3.98 (t, 2H, OCH_2_, *J* = 6.4), 2.34 (t, 2H, CH_2_COOEt, *J* = 7.2), 1.84 (m, 6H, CH_2_), 1.71 (m, 2H, CH_2_), 1.52 (m, 4H, CH_2_), 1.36 (m, 8H, CH_2_), 1.26 (t, 3H, CH_3_, *J* = 7.2), 0.91 (t, 3H, CH_3_, *J* = 7.2), 0.90 (t, 3H, CH_3_, *J* = 7.2); ^13^C NMR (100 MHz, CDCl_3_): δ = 173.8 (C_q_, C=O), 167.9, 167.2 (C_q_, N=C–S), 159.3 (C_q_, C–OCH_2_), 154.0, 153.3 (C_q_, C–OC_6_H_13_), 133.2, 132.6, 132.4 (C_t_), 129.4 (C_t_), 129.1 (C_q_), 127.9 (C_t_), 126.8, 125.8 (C_q_), 117.0, 116.8 (C_t_), 115.4, 115.2 (C_q_), 114.6 (C_t_), 112.9 (C_q_), 95.6, 94.0, 89.2, 84.7 (C≡C), 69.8, 69.7, 67.8 (OCH_2_), 60.4 (COOCH_2_CH_3_), 34.4 (CH_2_COOEt), 31.7, 29.5, 29.4, 29.0, 25.9, 25.8, 24.8, 22.8 (CH_2_), 14.4, 14.2 (CH_3_); EA: Calc. for C_50_H_55_BrN_2_O_5_S: C 68.56, H 6.33, N 3.20, S 3.66; Found: C 68.13, H 6.09, N 3.24, S 3.55; FD MS: *m/z* [%]: 875.7 (100, [M+2]^+^); 873.7 (77, M^+^).

**2-{4-[4-{4-[6-(Ethoxycarbonyl)hexyloxy]phenylethynyl}-2,5-bis(hexyloxy)phenyl]­ethynylphenyl}-5-(4-bromophenyl)-1,3,4-thiadiazole (3c)** Hexane/EtOAc = 6/1 (*R_f_* = 0.35), yellow solid; yield 0.33 g (66%). ^1^H NMR (400 MHz, CDCl_3_): δ = 7.99 (2H, AA’BB’), 7.88 (2H, AA’BB’), 7.64 (4H, AA’BB’), 7.46 (2H, AA’BB’), 7.02 (s, 1H), 7.01 (s, 1H), 6.86 (2H, AA’BB’), 4.13 (q, 2H, COOCH_2_CH_3_, *J* = 7.2), 4.04 (t, 2H, OCH_2_, *J* = 6.4), 4.03 (t, 2H, OCH_2_, *J* = 6.4), 3.97 (t, 2H, OCH_2_, *J* = 6.4), 2.32 (t, 2H, CH_2_COOEt, *J* = 7.2), 1.84 (m, 6H, CH_2_), 1.67 (m, 2H, CH_2_), 1.59–1.35 (m, 16H, CH_2_), 1.26 (t, 3H, CH_3_, ^3^*J*_HH_ = 7.2), 0.91 (t, 3H, CH_3_, *J* = 7.2), 0.90 (t, 3H, CH_3_, *J* = 7.2); ^13^C NMR (100 MHz, CDCl_3_): δ = 173.9 (C_q_, C=O), 167.9, 167.2 (C_q_, N=C–S), 159.4 (C_q_, C–OCH_2_), 154.0, 153.5 (C_q_, C–OC_6_H_13_), 133.2, 132.6, 132.4 (C_t_), 129.4 (C_t_), 129.1 (C_q_), 127.9 (C_t_), 126.8, 125.8 (C_q_), 117.0, 116.8 (C_t_), 115.4, 115.2 (C_q_), 114.6 (C_t_), 112.8 (C_q_), 95.6, 94.0, 89.2, 84.7 (C≡C), 69.8, 69.7, 68.0 (OCH_2_), 60.4 (COOCH_2_CH_3_), 34.4 (CH_2_COOEt), 31.8, 29.5, 29.4, 29.1, 29.0, 25.90, 25.88, 25.0, 22.82, 22.80 (CH_2_), 14.4, 14.2 (CH_3_); EA: Calc. for C_51_H_57_BrN_2_O_5_S: C 68.83, H 6.46, N 3.15, S 3.60; Found: C 68.31, H 6.19, N 3.22, S 3.50; FD MS: *m/z* [%]: 889.8 (100, [M+2]^+^); 887.9 (40, M^+^).

**2-{4-[4-{4-[7-(Ethoxycarbonyl)heptyloxy]phenylethynyl}-2,5-bis(hexyloxy)phenyl]­ethynylphenyl}-5-(4-bromophenyl)-1,3,4-thiadiazole (3d)** Hexane/EtOAc = 6/1 (*R_f_* = 0.35), yellow solid; yield 0.32 g (51%). ^1^H NMR (400 MHz, CDCl_3_): δ = 7.99 (2H, AA’BB’), 7.88 (2H, AA’BB’), 7.64 (4H, AA’BB’), 7.46 (2H, AA’BB’), 7.02 (s, 1H), 7.01 (s, 1H), 6.86 (2H, AA’BB’), 4.10 (q, 2H, COOCH_2_CH_3_, *J* = 7.2), 4.03 (t, 2H, OCH_2_, *J* = 6.4), 4.02 (t, 2H, OCH_2_, *J* = 6.4), 3.97 (t, 2H, OCH_2_, *J* = 6.4), 2.30 (t, 2H, CH_2_COOEt, *J* = 7.2), 1.90–1.75 (m, 6H, CH_2_), 1.64 (m, 2H, CH_2_), 1.60–1.44 (m, 12H, CH_2_), 1.36 (m, 12H, CH_2_), 1.26 (t, 3H, CH_3_, ^3^*J*_HH_ = 7.2), 0.91 (t, 3H, CH_3_, ^3^*J*_HH_ = 7.2), 0.90 (t, 3H, CH_3_, ^3^*J*_HH_ = 7.2); ^13^C NMR (100 MHz, CDCl_3_): δ = 174.0 (C_q_, C=O), 167.9, 167.2 (C_q_, N=C–S), 159.4 (C_q_, C–OCH_2_), 154.0, 153.5 (C_q_, C–OC_6_H_13_), 133.2, 132.6, 132.2 (C_t_), 129.4 (C_t_), 129.1 (C_q_), 127.9 (C_t_), 126.8, 125.8 (C_q_), 117.0, 116.7 (C_t_), 115.3, 115.2 (C_q_), 114.6 (C_t_), 112.8 (C_q_), 95.6, 94.0, 89.2, 84.6 (C≡C), 69.8, 69.7, 68.1 (OCH_2_), 60.4 (COOCH_2_CH_3_), 34.5 (CH_2_COOEt), 31.8, 29.5, 29.4, 29.3, 29.18, 29.16, 26.0, 25.9, 25.0, 22.81, 22.80 (CH_2_), 14.4, 14.2 (CH_3_); FD MS: *m/z* [%]:903.5 (100, [M+2]^+^); 901.5 (83, M^+^).

**2-{4-[4-{4-[10-(Ethoxycarbonyl)decyloxy]phenylethynyl}-2,5-bis(hexyloxy)phenyl]­ethynylphenyl}-5-(4-bromophenyl)-1,3,4-thiadiazole (3e)** Hexane/EtOAc = 6/1 (*R_f_* = 0.30), yellow solid; yield 0.46 g (58%). ^1^H NMR (400 MHz, CDCl_3_): δ = 8.00 (2H, AA’BB’), 7.89 (2H, AA’BB’), 7.64 (4H, AA’BB’), 7.46 (2H, AA’BB’), 7.02 (s, 1H), 7.01 (s, 1H), 6.87 (2H, AA’BB’), 4.12 (q, 2H, COOCH_2_CH_3_, *J* = 7.2), 4.044 (t, 2H, OCH_2_, *J* = 6.4), 4.036 (t, 2H, OCH_2_, *J* = 6.4), 3.97 (t, 2H, OCH_2_, *J* = 6.4), 2.29 (t, 2H, CH_2_COOEt, *J* = 7.2), 1.90–1.74 (m, 6H, CH_2_), 1.60–1.50 (m, 8H, CH_2_), 1.40–1.28 (m, 18H, CH_2_), 1.26 (t, 3H, CH_3_, ^3^*J*_HH_ = 7.2), 0.91 (t, 3H, CH_3_, *J* = 7.2), 0.90 (t, 3H, CH_3_, *J* = 7.2); ^13^C NMR (100 MHz, CDCl_3_): δ = 174.1 (C_q_, C=O), 167.9, 167.2 (C_q_, N=C–S), 159.5 (C_q_, C–OCH_2_), 154.0, 153.6 (C_q_, C–OC_6_H_13_), 133.2, 132.6, 132.3 (C_t_), 129.4 (C_t_), 129.2 (C_q_), 127.9 (C_t_), 126.8, 125.8 (C_q_), 117.1, 116.9 (C_t_), 115.4 (C_q_), 114.7 (C_t_), 112.9 (C_q_), 95.7, 94.0, 89.3, 84.7 (C≡C), 69.9, 69.7, 68.2 (OCH_2_), 60.3 (COOCH_2_CH_3_), 34.5 (CH_2_COOEt), 31.8, 29.6, 29.5, 29.39, 29.35, 29.28, 26.2, 25.9, 25.1, 22.8 (CH_2_), 14.4, 14.2 (CH_3_); EA: Calc. for C_55_H_65_BrN_2_O_5_S: C 69.82, H 6.92, N 2.96, S 3.39; Found: C 69.88, H 6.77, N 3.25, S 3.22; FD MS: *m/z* [%]: 946.2 (100, M^+^).

Analytical data of V-shaped mesogens **1b**–**e** and **2a**–**g**.

**2,5-Bis{4-[4-{4-[5-(ethoxycarbonyl)pentyloxy]phenylethynyl}-2,5-bis(hexyloxy)phenyl]­ethynylphenyl}-1,3,4-thiadiazole (1b)** Hexane/EtOAc = 6/1 (*R_f_* = 0.1), yellow solid; yield 95 mg (54%). ^1^H NMR (400 MHz, CDCl_3_): δ = 8.01 (4H, AA’BB’), 7.64 (4H, AA’BB’), 7.46 (4H, AA’BB’), 7.02 (s, 2H), 7.01 (s, 2H), 6.86 (4H, AA’BB’), 4.13 (q, 4H, COOCH_2_CH_3_, *J* = 7.2), 4.05 (t, 4H, OCH_2_, *J* = 6.4), 4.04 (t, 4H, OCH_2_, *J* = 6.4), 3.98 (t, 4H, OCH_2_, *J* = 6.4), 2.34 (t, 4H, CH_2_COOEt, *J* = 7.2), 1.85 (m, 12H, CH_2_), 1.71 (m, 4H, CH_2_), 1.55 (m, 12H, CH_2_), 1.37 (m, 16H, CH_2_), 1.26 (t, 6H, CH_3_, *J* = 7.2), 0.91 (t, 6H, CH_3_, *J* = 7.2), 0.90 (t, 6H, CH_3_, *J* = 7.2); ^13^C NMR (100 MHz, CDCl_3_): δ = 173.8 (C_q_, C=O), 167.9 (C_q_, N=C–S), 159.3 (C_q_, C–OCH_2_), 154.0, 153.5 (C_q_, C–OC_6_H_13_), 133.2, 132.3 (C_t_), 129.6 (C_q_), 127.9 (C_t_), 126.7 (C_q_), 117.0, 116.8 (C_t_), 115.4, 115.2 (C_q_), 114.6 (C_t_), 112.9 (C_q_), 95.6, 94.1, 89.2, 84.7 (C≡C), 69.8, 69.7, 67.8 (OCH_2_), 60.4 (COOCH_2_CH_3_), 34.4 (CH_2_COOEt), 31.8, 29.46, 29.45, 29.0, 25.9, 25.8, 24.8, 22.81, 22.80 (CH_2_); 14.4, 14.2 (CH_3_); EA: Calc. for C_86_H_102_N_2_O_10_S: C 76.19, H 7.58, N 2.07, S 2.37; Found: C 76.21, H 7.40, N 2.17, S 2.31; FD MS: *m/z* [%]: 1355.7 (45, [M+2]^+^); 1354.7 (78, [M+1]^+^); 1353.7 (100, M^+^).

**2,5-Bis{4-[4-{4-[6-(ethoxycarbonyl)hexyloxy]phenylethynyl}-2,5-bis(hexyloxy)phenyl]­ethynylphenyl}-1,3,4-thiadiazole (1c)** Hexane/EtOAc = 7/1 (*R_f_* = 0.1), yellow solid; yield 100 mg (68%). ^1^H NMR (400 MHz, CDCl_3_): δ = 8.01 (4H, AA’BB’), 7.64 (4H, AA’BB’), 7.46 (4H, AA’BB’), 7.02 (s, 2H), 7.01 (s, 2H), 6.86 (4H, AA’BB’), 4.13 (q, 4H, COOCH_2_CH_3_, *J* = 7.2), 4.05 (t, 4H, OCH_2_, *J* = 6.4), 4.04 (t, 4H, OCH_2_, *J* = 6.4), 3.97 (t, 4H, OCH_2_, *J* = 6.4), 2.32 (t, 4H, CH_2_COOEt, *J* = 7.2), 1.87–1.78 (m, 12H, CH_2_), 1.67 (m, 4H, CH_2_), 1.52 (m, 16H, CH_2_), 1.37 (m, 16H, CH_2_), 1.26 (t, 6H, CH_3_, *J* = 7.2), 0.91 (t, 6H, CH_3_, *J* = 7.2), 0.90 (t, 6H, CH_3_, *J* = 7.2); ^13^C NMR (100 MHz, CDCl_3_): δ = 173.9 (C_q_, C=O), 167.8 (C_q_, N=C–S), 159.4 (C_q_, C–OCH_2_), 154.0, 153.5, (C_q_, C–OC_6_H_13_), 133.2, 132.3 (C_t_), 129.6 (C_q_), 127.9 (C_t_), 126.7 (C_q_), 117.0, 116.8 (C_t_), 115.4, 115.2 (C_q_), 114.6 (C_t_), 112.9 (C_q_), 95.6, 94.1, 89.2, 84.7 (C≡C), 69.8, 69.7, 68.0 (OCH_2_), 60.4 (COOCH_2_CH_3_), 34.4 (CH_2_COOEt), 31.8, 29.5, 29.4, 29.2, 29.0, 25.90, 25.88, 25.0, 22.82, 22.80 (CH_2_), 14.4, 14.2 (CH_3_); EA: Calc. for C_88_H_106_N_2_O_10_S: C 76.38, H 7.72, N 2.02, S 2.32; Found: C 75.90, H 7.29, N 2.23, S 2.17; FD MS: *m/z* [%]: 1382.7 (93, [M+1]^+^); 1381.7 (100, M^+^).

**2,5-Bis{4-[4-{4-[7-(ethoxycarbonyl)heptyloxy]phenylethynyl}-2,5-bis(hexyloxy)phenyl]­ethynylphenyl}-1,3,4-thiadiazole (1d)** Hexane/EtOAc = 6/1 (*R_f_* = 0.1), yellow solid; yield 75 mg (50%). ^1^H NMR (400 MHz, CDCl_3_): δ = 8.01 (4H, AA’BB’), 7.64 (4H, AA’BB’), 7.46 (4H, AA’BB’), 7.02 (s, 2H), 7.01 (s, 2H), 6.86 (4H, AA’BB’), 4.13 (q, 4H, COOCH_2_CH_3_, ^3^*J*_HH_ = 7.2), 4.05 (t, 4H, OCH_2_, ^3^*J*_HH_ = 6.4), 4.04 (t, 4H, OCH_2_, ^3^*J*_HH_ = 6.4), 3.97 (t, 4H, OCH_2_, ^3^*J*_HH_ = 6.4), 2.30 (t, 4H, CH_2_–COOEt, ^3^*J*_HH_ = 7.2), 1.90–1.75 (m, 12H, CH_2_), 1.68–1.32 (m, 42H, CH_2_), 1.26 (t, 6H, CH_3_, ^3^*J*_HH_ = 7.2), 0.91 (t, 6H, CH_3_, ^3^*J*_HH_ = 7.2), 0.90 (t, 6H, CH_3_, ^3^*J*_HH_ = 7.2); ^13^C NMR (100 MHz, CDCl_3_): δ = 174.0 (C_q_, C=O), 167.8 (C_q_, N=C–S), 159.4 (C_q_, C–OCH_2_), 154.0, 153.5 (C_q_, C–OC_6_H_13_), 133.2, 132.4 (C_t_), 129.6 (C_q_), 128.0 (C_t_), 126.7 (C_q_), 117.0, 116.8 (C_t_), 115.4, 115.2 (C_q_), 114.6 (C_t_), 112.9 (C_q_), 95.6, 94.1, 89.2, 84.6 (C≡C), 69.8, 69.7, 68.1 (OCH_2_), 60.4 (COOCH_2_CH_3_), 34.5 (CH_2_COOEt), 31.8, 29.5, 29.4, 29.3, 29.2, 26.9, 25.9, 25.0, 22.82, 22.80 (CH_2_), 14.4, 14.2 (CH_3_); FD-MS**:** *m/z* [%]: 1410.5 (63, [M+1]^+^); 1409.5 (67, M^+^); 705.0 (100, M^2+^).

**2,5-Bis{4-[4-{4-[10-(ethoxycarbonyl)decyloxy]phenylethynyl}-2,5-bis(hexyloxy)phenyl]­ethynylphenyl}-1,3,4-thiadiazole (1e)** Hexane/EtOAc = 13/1 (*R_f_* = 0.07), yellow solid; yield 80 mg (51%). ^1^H NMR (400 MHz, CDCl_3_): δ = 8.01 (4H, AA’BB’), 7.65 (4H, AA’BB’), 7.46 (4H, AA’BB’), 7.02 (s, 2H), 7.01 (s, 2H), 6.87 (4H, AA’BB’), 4.13 (q, 4H, COOCH_2_CH_3_, *J* = 7.2), 4.05 (t, 4H, OCH_2_, *J* = 6.4), 4.04 (t, 4H, OCH_2_, *J* = 6.4), 3.97 (t, 4H, OCH_2_, *J* = 6.4), 2.29 (t, 4H, CH_2_COOEt, *J* = 7.2), 1.90–1.75 (m, 12H, CH_2_), 1.66–1.51 (m, 16H, CH_2_), 1.50–1.30 (m, 36H, CH_2_), 1.26 (t, 6H, CH_3_, *J* = 7.2), 0.91 (t, 6H, CH_3_, *J* = 7.2), 0.90 (t, 6H, CH_3_, *J* = 7.2); ^13^C NMR (100 MHz, CDCl_3_): δ = 174.1 (C_q_, C=O), 167.8 (C_q_, N=C–S), 159.5 (C_q_, C–OCH_2_), 154.0, 153.5 (C_q_, C–OC_6_H_13_), 133.2, 132.4 (C_t_), 129.6 (C_q_), 128.0 (C_t_), 126.7 (C_q_), 117.0, 116.8 (C_t_), 115.3, 115.2 (C_q_), 114.6 (C_t_), 112.9 (C_q_), 95.6, 94.1, 89.2, 84.6 (C≡C), 69.8, 69.7, 68.2 (OCH_2_), 60.3 (COOCH_2_CH_3_), 34.5 (CH_2_COOEt), 31.8, 29.6, 29.52, 29.47, 29.45, 29.40, 29.34, 29.27, 26.1, 25.9, 25.1, 22.82, 22.80 (CH_2_), 14.4, 14.2 (CH_3_); EA: Calc. for C_96_H_122_N_2_O_10_S: C 77.07, H 8.22, N 1.87, S 2.14; Found: C 76.54, H 7.76, N 2.13, S 2.22; FD MS: *m/z* [%]: 1494.6 (100, [M+1]^+^); 1493.6 (93, M^+^).

**2-{4-[4-{4-[5-(Ethoxycarbonyl)pentyloxy]phenylethynyl}-2,5-bis(hexyloxy)phenyl]­ethynylphenyl}-5-{4-[4-{4-[4-(ethoxycarbonyl)butoxy]phenylethynyl}-2,5-bis(hexyloxy)phenyl]ethynylphenyl}-1,3,4-thiadiazole (2a)** Hexane/EtOAc = 7/1 (*R_f_* = 0.1), yellow solid; yield 87 mg (61%). ^1^H NMR (400 MHz, CDCl_3_): δ = 8.01 (4H, AA’BB’), 7.64 (4H, AA’BB’), 7.46 (4H, AA’BB’), 7.02 (s, 2H), 7.01 (s, 2H), 6.86 (4H, AA’BB’), 4.135 (q, 2H, COOCH_2_CH_3_, *J* = 7.2), 4.134 (q, 2H, COOCH_2_CH_3_, *J* = 7.2), 4.043 (t, 4H, OCH_2_, *J* = 6.4), 4.036 (t, 4H, OCH_2_, *J* = 6.4), 3.98 (m, 4H, OCH_2_), 2.39 (t, 2H, CH_2_COOEt, *J* = 7.2), 2.34 (t, 2H, CH_2_COOEt, *J* = 7.2), 1.85 (m, 12H, CH_2_), 1.71 (m, 2H, CH_2_), 1.55 (m, 12H, CH_2_), 1.37 (m, 16H, CH_2_), 1.262 (t, 3H, CH_3_, *J* = 7.2), 1.259 (t, 3H, CH_3_, *J* = 7.2), 0.91 (t, 6H, CH_3_, *J* = 7.2), 0.90 (t, 6H, CH_3_, *J* = 7.2); ^13^C NMR (100 MHz, CDCl_3_): δ = 173.8, 173.6 (C_q_, C=O), 167.9 (C_q_, N=C–S), 159.3, 159.2 (C_q_, C–OCH_2_), 154.0, 153.5, (C_q_, C–OC_6_H_13_), 133.2, 132.3 (C_t_), 129.6 (C_q_), 127.9 (C_t_), 126.7 (C_q_), 117.0, 116.8 (C_t_), 115.5, 115.4, 115.2 (C_q_), 114.6 (C_t_), 112.9 (C_q_), 95.6, 94.1, 89.2, 84.7 (C≡C), 69.8, 69.7, 67.8, 67.6 (OCH_2_), 60.5, 60.4 (COOCH_2_CH_3_), 34.4, 34.1 (CH_2_COOEt), 31.8, 29.5, 29.0, 28.8, 25.9, 25.8, 24.8, 22.8, 21.7 (CH_2_), 14.4, 14.2 (CH_3_); EA: Calc. for C_85_H_100_N_2_O_10_S: C 76.09, H 7.51, N 2.09, S 2.39; Found: C 76.49, H 7.51, N 2.16, S 2.30; FD MS: *m/z* [%]:1340.7 (88, [M+1]^+^); 1339.7 (100, M^+^).

**2-{4-[4-{4-[6-(Ethoxycarbonyl)hexyloxy]phenylethynyl}-2,5-bis(hexyloxy)phenyl]­ethynylphenyl}-5-{4-[4-{4-[4-(ethoxycarbonyl)butoxy]phenylethynyl}-2,5-bis(hexyloxy)phenyl]ethynylphenyl}-1,3,4-thiadiazole (2b)** Hexane/EtOAc = 7/1 (*R_f_* = 0.1), yellow solid; yield 90 mg (60%). ^1^H NMR (400 MHz, CDCl_3_): δ = 8.01 (4H, AA’BB’), 7.64 (4H, AA’BB’), 7.46 (4H, AA’BB’), 7.02 (s, 2H), 7.01 (s, 2H), 6.86 (4H, AA’BB’), 4.14 (q, 2H, COOCH_2_CH_3_, *J* = 7.2), 4.13 (q, 2H, COOCH_2_CH_3_, *J* = 7.2), 4.05 (t, 4H, OCH_2_, *J* = 6.4), 4.04 (t, 4H, OCH_2_, *J* = 6.4), 3.99 (m, 4H, OCH_2_), 2.40 (t, 2H, CH_2_COOEt, *J* = 7.2), 2.32 (t, 2H, CH_2_COOEt, *J* = 7.2), 1.85 (m, 14H, CH_2_), 1.67 (m, 2H, CH_2_), 1.52 (m, 12H, CH_2_), 1.37 (m, 16H, CH_2_), 1.264 (t, 3H, CH_3_, *J* = 7.2), 1.258 (t, 3H, CH_3_, *J* = 7.2), 0.91 (t, 6H, CH_3_, *J* = 7.2), 0.90 (t, 6H, CH_3_, *J* = 7.2); ^13^C NMR (100 MHz, CDCl_3_): δ = 173.8, 173.6 (C_q_, C=O), 167.9 (C_q_, N=C–S), 159.3, 159.2 (C_q_, C–OCH_2_), 154.0, 153.5, (C_q_, C–OC_6_H_13_), 133.2, 132.3 (C_t_), 129.6 (C_q_), 127.9 (C_t_), 126.7 (C_q_), 117.0, 116.8 (C_t_), 115.5, 115.4, 115.2 (C_q_), 114.6 (C_t_), 112.9 (C_q_), 95.6, 94.1, 89.2, 84.7 (C≡C), 69.8, 69.7, 67.8, 67.6 (OCH_2_), 60.5, 60.4 (COOCH_2_CH_3_), 34.4, 34.1 (CH_2_COOEt), 31.8, 29.5, 29.0, 28.8, 25.9, 25.8, 24.8, 22.8, 21.7 (CH_2_), 14.4, 14.2 (CH_3_). EA: Calc. for C_86_H_102_N_2_O_10_S: C 76.19, H 7.58, N 2.07, S 2.37; Found: C 75.91, H 7.87, N 1.95, S 2.25. FD MS: *m/z* [%]:1355.3 (90, M^+^); 1354.3 (100).

**2-{4-[4-{4-[7-(Ethoxycarbonyl)heptyloxy]phenylethynyl}-2,5-bis(hexyloxy)phenyl]­ethynylphenyl}-5-{4-[4-{4-[4-(ethoxycarbonyl)butoxy]phenylethynyl}-2,5-bis­(hexyloxy)phenyl]ethynylphenyl}-1,3,4-thiadiazole (2c)** Hexane/EtOAc = 7/1 (*R_f_* = 0.1), yellow solid; yield 91 mg (63%). ^1^H NMR (400 MHz, CDCl_3_): δ = 8.01 (4H, AA’BB’), 7.64 (4H, AA’BB’), 7.46 (4H, AA’BB’), 7.02 (s, 2H), 7.01 (s, 2H), 6.86 (4H, AA’BB’), 4.14 (q, 2H, COOCH_2_CH_3_, *J* = 7.2), 4.13 (q, 2H, COOCH_2_CH_3_, *J* = 7.2), 4.05 (t, 4H, OCH_2_, *J* = 6.4), 4.04 (t, 4H, OCH_2_, *J* = 6.4), 3.98 (m, 4H, OCH_2_), 2.39 (t, 2H, CH_2_COOEt, *J* = 7.2), 2.30 (t, 2H, CH_2_COOEt, *J* = 7.2), 1.90–1.76 (m, 14H, CH_2_), 1.68–1.51 (m, 10H, CH_2_), 1.50 – 1.32 (m, 22H, CH_2_), 1.264 (t, 3H, CH_3_, *J* = 7.2), 1.258 (t, 3H, CH_3_, *J* = 7.2), 0.91 (t, 6H, CH_3_, *J* = 7.2), 0.90 (t, 6H, CH_3_, *J* = 7.2); ^13^C NMR (100 MHz, CDCl_3_): δ = 174.0, 173.6 (C_q_, C=O), 167.8 (C_q_, N=C–S), 159.4, 159.2 (C_q_, C–OCH_2_), 154.0, 153.5 (C_q_, C–OC_6_H_13_), 133.2, 132.4 (C_t_), 129.6 (C_q_), 128.0 (C_t_), 126.7 (C_q_), 117.0, 116.8 (C_t_), 115.5, 115.4, 115.21, 115.18 (C_q_), 114.62, 114.61 (C_t_), 112.89, 112.87 (C_q_), 95.6, 95.5, 94.1, 89.2, 84.7, 84.6 (C≡C), 69.8, 69.7, 68.1, 67.6 (OCH_2_), 60.5, 60.4 (COOCH_2_CH_3_), 34.5, 34.1 (CH_2_COOEt), 31.8, 29.5, 29.3, 29.2, 28.7, 26.0, 25.9, 25.0, 22.82, 22.80, 21.8 (CH_2_), 14.4, 14.2 (CH_3_); EA: Calc. for C_87_H_104_N_2_O_10_S: C 76.28, H 7.65, N 2.05, S 2.34; Found: C 75.81, H 7.37, N 2.23, S 2.23; FD MS: *m/z* [%]: 1368.6 (90, [M+1]^+^); 1367.6 (100, M^+^).

**2-{4-[4-{4-[5-(Ethoxycarbonyl)pentyloxy]phenylethynyl}-2,5-bis(hexyloxy)phenyl]­ethynylphenyl}-5-{4-[4-{4-[6-(ethoxycarbonyl)hexyloxy]phenylethynyl}-2,5-bis­(hexyloxy)phenyl]ethynylphenyl}-1,3,4-thiadiazole (2d)** Hexane/EtOAc = 7/1 (*R_f_* = 0.1), yellow solid; yield 95 mg (65%). ^1^H NMR (400 MHz, CDCl_3_): δ = 8.01 (4H, AA’BB’), 7.64 (4H, AA’BB’), 7.46 (4H, AA’BB’), 7.02 (s, 2H), 7.01 (s, 2H), 6.85 (4H, AA’BB’), 4.133 (q, 2H, COOCH_2_CH_3_, *J* = 7.2), 4.128 (q, 2H, COOCH_2_CH_3_, *J* = 7.2), 4.042 (t, 4H, OCH_2_, *J* = 6.4), 4.035 (t, 4H, OCH_2_, *J* = 6.4), 3.98 (t, 2H, OCH_2_, *J* = 6.4), 3.97 (t, 2H, OCH_2_, *J* = 6.4), 2.34 (t, 2H, CH_2_COOEt, *J* = 7.2), 2.31 (t, 2H, CH_2_-COOEt, *J* = 7.2), 1.90–1.78 (m, 12H, CH_2_), 1.68 (m, 4H, CH_2_), 1.60–1.47 (m, 12H, CH_2_), 1.37 (m, 16H, CH_2_), 1.258 (t, 6H, CH_3_, *J* = 7.2), 1.255 (t, 6H, CH_3_, *J* = 7.2), 0.91 (t, 6H, CH_3_, *J* = 7.2), 0.90 (t, 6H, CH_3_, *J* = 7.2); ^13^C NMR (100 MHz, CDCl_3_): δ = 173.9, 173.8 (C_q_, C=O), 167.8 (C_q_, N=C–S), 159.39, 159.33 (C_q_, C–OCH_2_), 154.0, 153.5 (C_q_, C–OC_6_H_13_), 133.2, 132.3 (C_t_), 129.6 (C_q_), 127.9 (C_t_), 126.7 (C_q_), 117.0, 116.8 (C_t_), 115.44, 115.39, 115.2 (C_q_), 114.6 (C_t_), 112.9 (C_q_), 95.6, 94.1, 89.2, 84.7 (C≡C), 69.8, 69.7, 68.0, 67.8 (OCH_2_), 60.43, 60.38 (COOCH_2_CH_3_), 34.4, 34.1 (CH_2_COOEt), 31.8, 29.5, 29.2, 29.0, 25.9, 25.8, 25.0, 24.8, 22.83, 22.81, 21.7 (CH_2_), 14.4, 14.2 (CH_3_); EA: Calc. for C_87_H_104_N_2_O_10_S: C 76.28, H 7.65, N 2.05, S 2.34; Found: C 76.12, H 7.55, N 2.11, S 2.18; FD MS: *m/z* [%]: 1368.6 (88, [M+1]^+^); 1367.8 (100, M^+^).

**2-{4-[4-{4-[7-(Ethoxycarbonyl)heptyloxy]phenylethynyl}-2,5-bis(hexyloxy)phenyl]­ethynylphenyl}-5-{4-[4-{4-[6-(ethoxycarbonyl)hexyloxy]phenylethynyl}-2,5-bis­(hexyloxy)phenyl]ethynylphenyl}-1,3,4-thiadiazole (2e)** Hexane/EtOAc = 7/1 (*R_f_* = 0.1), yellow solid; yield 100 mg (68%). ^1^H NMR (400 MHz, CDCl_3_): δ = 8.01 (4H, AA’BB’), 7.64 (4H, AA’BB’), 7.46 (4H, AA’BB’), 7.02 (s, 2H), 7.01 (s, 2H), 6.86 (4H, AA’BB’), 4.13 (q, 4H, COOCH_2_CH_3_, *J* = 7.2), 4.05 (t, 4H, OCH_2_, *J* = 6.4), 4.04 (t, 4H, OCH_2_, *J* = 6.4), 3.970 (t, 2H, OCH_2_, *J* = 6.4), 3.968 (t, 2H, OCH_2_, *J* = 6.4), 2.32 (t, 2H, CH_2_COOEt, *J* = 7.6), 2.30 (t, 2H, CH_2_COOEt, *J* = 7.6), 1.90–1.76 (m, 12H, CH_2_), 1.60–1.32 (m, 34H, CH_2_), 1.26 (t, 6H, CH_3_, *J* = 7.2), 0.91 (t, 6H, CH_3_, *J* = 7.2), 0.90 (t, 6H, CH_3_, *J* = 7.2); ^13^C NMR (100 MHz, CDCl_3_): δ = 174.0, 173.9 (C_q_, C=O), 167.8 (C_q_, N=C–S), 159.41, 159.38 (C_q_, C–OCH_2_), 154.0, 153.5 (C_q_, C–OC_6_H_13_), 133.2, 132.3 (C_t_), 129.6 (C_q_), 127.9 (C_t_), 126.7 (C_q_), 117.0, 116.8 (C_t_), 115.39, 115.36, 115.2 (C_q_), 114.6 (C_t_), 112.9 (C_q_), 95.6, 94.1, 89.2, 84.7 (C≡C), 69.8, 69.7, 68.1, 68.0 (OCH_2_), 60.37, 60.35 (COOCH_2_CH_3_), 34.5, 34.4 (CH_2_COOEt), 31.8, 29.5, 29.3, 29.2, 29.0, 28.7, 26.0, 25.9, 25.02, 25.00, 22.82, 22.80 (CH_2_), 14.4, 14.2 (CH_3_); EA: Calc. for C_89_H_108_N_2_O_10_S: C 76.47, H 7.79, N 2.00, S 2.29; Found: C 76.17, H 7.73, N 2.18, S 2.29; FD MS: *m/z* [%]: 1396.5 (100, [M+1]^+^); 1395.5 (94, M^+^).

**2-{4-[4-{4-[10-(Ethoxycarbonyl)decyloxy]phenylethynyl}-2,5-bis(hexyloxy)phenyl]­ethynylphenyl}-5-{4-[4-{4-[4-(ethoxycarbonyl)butoxy]phenylethynyl}-2,5-bis-(hexyloxy)phenyl]ethynylphenyl}-1,3,4-thiadiazole (2f)** Hexane/EtOAc = 7/1 (*R_f_* = 0.1), yellow solid; yield 100 mg (67%). ^1^H NMR (400 MHz, CDCl_3_): δ = 8.01 (4H, AA’BB’), 7.64 (4H, AA’BB’), 7.46 (4H, AA’BB’), 7.02 (s, 2H), 7.01 (s, 2H), 6.86 (4H, AA’BB’), 4.14 (q, 2H, COOCH_2_CH_3_, *J* = 7.2), 4.12 (q, 2H, COOCH_2_CH_3_, *J* = 7.2), 4.05 (t, 4H, OCH_2_, *J* = 6.4), 4.04 (t, 4H, OCH_2_, *J* = 6.4), 3.97 (m, 4H, OCH_2_), 2.39 (t, 2H, CH_2_COOEt, *J* = 7.2), 2.29 (t, 2H, CH_2_COOEt, *J* = 7.2), 1.90–1.74 (m, 14H, CH_2_), 1.66–1.50 (m, 12H, CH_2_), 1.50–1.30 (m, 26H, CH_2_), 1.26 (t, 3H, CH_3_, *J* = 7.2), 1.25 (t, 3H, CH_3_, *J* = 7.2), 0.91 (t, 6H, CH_3_, *J* = 7.2), 0.90 (t, 6H, CH_3_, *J* = 7.2); ^13^C NMR (100 MHz, CDCl_3_): δ = 174.0, 173.6 (C_q_, C=O), 167.8 (C_q_, N=C–S), 159.4, 159.2 (C_q_, C–OCH_2_), 154.0, 153.5 (C_q_, C–OC_6_H_13_), 133.2, 132.3 (C_t_), 129.6 (C_q_), 127.9 (C_t_), 126.7 (C_q_), 117.0, 116.8 (C_t_), 115.5, 115.3, 115.23, 115.18 (C_q_), 114.63, 114.60 (C_t_), 112.89, 112.86 (C_q_), 95.6, 95.5, 94.1, 89.2, 84.7, 84.6 (C≡C), 69.8, 69.7, 68.2, 67.6 (OCH_2_), 60.5, 60.3 (COOCH_2_CH_3_), 34.5, 34.1 (CH_2_COOEt), 31.8, 29.6, 29.51, 29.50, 29.46, 29.44, 29.38, 29.33, 29.27, 28.7, 26.1, 25.9, 25.1, 22.81, 22.79, 21.8 (CH_2_), 14.4, 14.2 (CH_3_); EA: Calc. for C_90_H_110_N_2_O_10_S: C 76.56, H 7.85, N 1.98, S 2.27; Found: C 76.38, H 7.65, N 2.36, S 2.08; FD MS: *m/z* [%]: 1411.3 (97, M^+^); 1410.3 (100).

**2-{4-[4-{4-[10-(Ethoxycarbonyl)decyloxy]phenylethynyl}-2,5-bis(hexyloxy)phenyl]­ethynylphenyl}-5-{4-[4-{4-[7-(ethoxycarbonyl)heptyloxy]phenylethynyl}-2,5-bis­(hexyloxy)phenyl]ethynylphenyl}-1,3,4-thiadiazole (2g)** Hexane/EtOAc = 10/1 (*R_f_* = 0.1), yellow solid; yield 88 mg (64%). ^1^H NMR (400 MHz, CDCl_3_): δ = 8.01 (4H, AA’BB’), 7.65 (4H, AA’BB’), 7.46 (4H, AA’BB’), 7.02 (s, 2H), 7.01 (s, 2H), 6.86 (4H, AA’BB’), 4.13 (q, 2H, COOCH_2_CH_3_, *J* = 7.2), 4.12 (q, 2H, COOCH_2_CH_3_, *J* = 7.2), 4.05 (t, 4H, OCH_2_, *J* = 6.4), 4.04 (t, 4H, OCH_2_, *J* = 6.4), 3.97 (t, 4H, OCH_2_, *J* = 6.4), 2.30 (t, 2H, CH_2_COOEt, *J* = 7.2), 2.29 (t, 2H, CH_2_COOEt, *J* = 7.2), 1.90–1.74 (m, 12H, CH_2_), 1.68–1.28 (m, 46H, CH_2_), 1.258 (t, 3H, CH_3_, *J* = 7.2), 1.255 (t, 3H, CH_3_, *J* = 7.2), 0.91 (t, 6H, CH_3_, *J* = 7.2), 0.90 (t, 6H, CH_3_, *J* = 7.2); ^13^C NMR (100 MHz, CDCl_3_): δ = 174.1, 174.0 (C_q_, C=O), 167.8 (C_q_, N=C–S), 159.4 (C_q_, C–OCH_2_), 154.0, 153.6 (C_q_, C–OC_6_H_13_), 133.2, 132.4 (C_t_), 129.6 (C_q_), 128.0 (C_t_), 126.7 (C_q_), 117.2, 116.8 (C_t_), 115.4, 115.2 (C_q_), 114.6 (C_t_), 112.9 (C_q_), 95.6, 94.1, 89.2, 84.7 (C≡C), 69.8, 69.7, 68.2, 68.1 (OCH_2_), 60.36, 60.32 (COOCH_2_CH_3_), 34.54, 34.48 (CH_2_COOEt), 31.8, 29.63, 29.50, 29.47, 29.45, 29.40, 29.34, 29.28, 29.19, 26.2, 26.0, 25.9, 25.12, 25.03, 22.82, 22.81 (CH_2_), 14.4, 14.2 (CH_3_). EA: Calc. for C_93_H_116_N_2_O_10_S: C 76.82, H 8.04, N 1.93, S 2.21; Found: C 76.62, H 8.09, N 1.89, S 2.03. FD MS: *m/z* [%]: 1453.2 (100, M^+^).

**Reference**

Wadsworth, H. J.; Widdowson, D. A.; Wilson, E.; Carroll, M. A. Radical trap in fluoridation of iodonium salt. WO 2005/061415 A1, July 7, 2005.
